# A qualitative case study of evaluation use in the context of a collaborative program evaluation strategy in Burkina Faso

**DOI:** 10.1186/s12961-016-0109-0

**Published:** 2016-05-26

**Authors:** Léna D’Ostie-Racine, Christian Dagenais, Valéry Ridde

**Affiliations:** Department of Psychology, University of Montreal, Pavillon Marie-Victorin, Room C355, P.O. Box 6128, Centre-ville Station, Montreal, Quebec H3C 3J7 Canada; Department of Social and Preventive Medicine, University of Montreal School of Public Health (ESPUM), Montreal, Canada; University of Montreal Public Health Research Institute (IRSPUM), Montreal, Canada

**Keywords:** Evaluation use, Utilization, Knowledge transfer, Program evaluation, Burkina Faso (West Africa), Healthcare, User fee exemption

## Abstract

**Background:**

Program evaluation is widely recognized in the international humanitarian sector as a means to make interventions and policies more evidence based, equitable, and accountable. Yet, little is known about the way humanitarian non-governmental organizations (NGOs) actually use evaluations.

**Methods:**

The current qualitative evaluation employed an instrumental case study design to examine evaluation use (EU) by a humanitarian NGO based in Burkina Faso. This organization developed an evaluation strategy in 2008 to document the implementation and effects of its maternal and child healthcare user fee exemption program. Program evaluations have been undertaken ever since, and the present study examined the discourses of evaluation partners in 2009 (n = 15) and 2011 (n = 17). Semi-structured individual interviews and one group interview were conducted to identify instances of EU over time. Alkin and Taut’s (Stud Educ Eval 29:1–12, 2003) conceptualization of EU was used as the basis for thematic qualitative analyses of the different forms of EU identified by stakeholders of the exemption program in the two data collection periods.

**Results:**

Results demonstrated that stakeholders began to understand and value the utility of program evaluations once they were exposed to evaluation findings and then progressively used evaluations over time. EU was manifested in a variety of ways, including instrumental and conceptual use of evaluation processes and findings, as well as the persuasive use of findings. Such EU supported planning, decision-making, program practices, evaluation capacity, and advocacy.

**Conclusions:**

The study sheds light on the many ways evaluations can be used by different actors in the humanitarian sector. Conceptualizations of EU are also critically discussed.

## Background

Humanitarian assistance organizations are increasing investing in program evaluation to enhance performance, practice and accountability [1–5]. Yet, ensuring knowledge derived from evaluation of humanitarian action, defined as the “*systematic and impartial examination of humanitarian action intended to draw lessons to improve policy and practice and enhance accountability*” [6], is actually used remains an important challenge [2, 4, 5, 7–9]. A common difficulty highlighted by Hallam [4] is that “*too often, humanitarian evaluations exist as a disconnected process, rather than becoming embedded as part of the culture and mindset of humanitarian organisations*”. The literature offers few examples of evaluation strategies that have been integrated into a humanitarian aid program, used effectively, and documented over time [10]. Rare also are studies that document the perspectives of both knowledge producers (e.g. evaluators) and intended users [10].

The present article examines evaluation use (EU) by HELP (*Hilfe zur Selbsthilfe e.V.*), a German humanitarian non-governmental organization (NGO) based in Burkina Faso that has developed an evaluation strategy now embedded into the country’s healthcare user fee exemption program [11–14]. The exemption program was implemented in Burkina Faso in part because of the country’s high rates of mortality and morbidity and its context of economic poverty, in which user fees undermine the accessibility of health services for many [13–16]. Especially in the Sahel region, where HELP implemented its user fee exemption program, maternal and infant rates of malnutrition, morbidity and mortality are exceedingly high, as shown in WHO’s 2014 statistical report [13, 14, 17]. HELP’s program is aimed at exempting indigents, pregnant and breastfeeding women, as well as children under five, from user fees [13]. Similar user fee subsidies or exemption programs had been attempted in different West African countries [18], but planning, implementation, and evaluation were frequently insufficient and often only partial [19, 20], and in general the measured impacts were smaller than expected [21]. Hence, while such exemption programs innovated upon previous practices in West Africa [22] and in some instances seemed promising [21], for a complex array of reasons, health sector deficiencies persisted and health indicators remained worrisome [21, 23, 24]. Thus, documenting and evaluating the implementation of innovative health financing programs has become increasingly necessary. West African decision-makers and practitioners have required empirical documentation on the processes and effects of user fee exemptions to ground their reflections, decisions and actions [18, 22, 23, 25, 26].

HELP had previously implemented an exemption program in Niger, which had been evaluated in 2007 at the request of its funding agency, the European Commission’s Humanitarian Aid and Civil Protection department (ECHO). The external evaluators were impressed by the HELP managers’ interest in evaluation findings and by their proactivity in implementing evaluation recommendations. Conscious that empirical evidence can support improvements in the humanitarian sector [23, 26], HELP managers consulted those same external evaluators while planning the Burkina Faso user fee exemption program, hoping to render it more evidence based. Together, the external evaluators and HELP managers developed an actual strategy for the evaluation, to be embedded within the user fee exemption program, and requested and were granted a specific budget for that evaluation strategy. Upon budget approval in 2008, HELP staff and the evaluators simultaneously developed both the Burkina Faso exemption program and the evaluation strategy aimed at documenting its implementation and effectiveness for purposes of accountability, program learning and improvement, and advocacy [8, 11]. Indeed, evaluating HELP’s exemption program as it evolved in Burkina Faso would provide opportunities for HELP and its partners to learn from and improve the exemption program. Resulting documentation could also be used to enhance HELP’s transparency and accountability and to facilitate its advocacy for equitable access to healthcare. Advocating for equitable access to healthcare was also one of ECHO’s objectives and hence was in line with its own mission. These were the main motives driving HELP decision-makers and their partners, including a principal evaluator, to develop the evaluation strategy.

Ridde et al. [12] have described in detail 12 of the studies undertaken by HELP as part of the evaluation strategy (Box 1). Stakeholders of the strategy, referred to in this article as evaluation partners (EPs), were primarily HELP’s exemption program staff and the external evaluators, but also included the Sahel regional health director (*directeur régional de la santé*, DRS), the district chief physicians (*médecins chefs de district*, MCDs), and representatives from ECHO, as well as advocacy partners, including a journalist and a representative of Amnesty International.

**Box 1** HELP evaluation studies from 2007 to 2011

**Table:** 

Studies on evaluation of effects
1. Assessment of effects on the population through a survey of a representative panel of households 2. Assessment of effects on health facilities using an interrupted time-series analysis 3. Assessment of the community context and of health centres (Centre de santé et de promotion sociale: CSPS) 4. Accounting study assessing the financial capacities of the community-based health centre management committees (comité de gestion: COGES) in the two districts by comparing data 12 months before and 6 months after the experiment 5. Appropriateness of prescriptions for children under the age of 5 years 6. Effectiveness of an indigent selection process assessed using a quantitative methodology 7. Assessment of effects on childbirth costs (*n* = 849) and particularly the estimation of excessive expenses for households 8. Effects on community participation and the empowerment of COGES members and women
Studies on assessment of processes and relevance
9. A process evaluation of user fees abolition for pregnant women and children under 5 years in two districts in Niger (West Africa) 10. User fees abolition policy in Niger: Comparing the under 5 years exemption implementation in two districts [27] 11. A case study into the times taken to reimburse procedures performed without payment, in a sample of ten CSPSs 12. A study on the costs of reimbursed procedures for children under the age of 5 years 13. A process assessment of an intervention’s progress, strengths and weaknesses, chances of continuing, merits and relevance 14. Analysis of relevance of an indigent selection process, performed during the same data collection for effects on community participation (see above) 15. Action-Research guided by Réseaux d’Accès aux Médicaments Essentiels (RAME) - Dori team: Quality of health services - Sebba team: maternal morbidity in the context of cost sharing, Soins obstétricaux néonataux d’urgence (SONU), and HELP’s exemption - RAME team: Treatment coverage at the Yalgado Ouedraogo Hospital in the context of the prepaid emergency kits 16. Assessment of health centre staff workload 17. Evaluation of HELP’s knowledge transfer strategy
Adapted from Ridde et al. [27]

Following an evaluability assessment of EU in Burkina Faso as part of the evaluation strategy described by Ridde et al. [12], it was clear the experiences of its EPs presented a rich opportunity to examine progressive EU over time [28]. More specifically, the present study is innovative in examining the different forms of EU in depth, using a diachronic approach to observe any variations in EU between 2009 and 2011 from the varied perspectives of the different EPs. EPs who had collaborated both on the Niger 2007 evaluation and on the evaluation strategy in Burkina Faso were able to discuss variations in EU between 2007 and 2011.

### Evaluation use

Traditionally, EU has been viewed solely as the use of evaluation findings, referring, for example, to the application of evaluation recommendations [29, 30]. In this view, after reading an evaluation report, staff in a humanitarian program aimed at alleviating malnutrition could, for example, strive to implement a recommendation to increase the supply of a given nutrient to toddlers of a given community. Current definitions of EU, however, include not only findings use but also process use, a term originally coined by Patton [31] to refer to the “*individual changes in thinking, attitudes, and behaviour, and program or organizational changes in procedures and culture that occur among those involved in evaluation as a result of the learning that occurs during the evaluation process*”. Patton [32] explained that process use could, for instance, manifest as “*infusing evaluative thinking into an organization’s culture*” [32], which might be seen in attempts to use more clear, specific, concrete and observable logic [31]. Humanitarian staff for the same nutritional program could, for example, learn during an evaluation process to specify clearer program objectives, beneficiary selection criteria, program actions and success indicators. Such process use could enhance shared understanding among them and potentially lead to program improvements and ultimately to lower rates of malnourishment. In the present study, we have attempted to attend to a broad spectrum of EUs by according no primacy to findings use over process use and by documenting unintended uses as well as uses that occurred over time in a cumulative or gradual manner.

The principal objective of the present study was to examine the diverse uses of evaluation findings and processes engendered by the evaluation strategy. A related objective was to examine whether any changes in EU occurred between 2009 and 2011. Hence, the focus was not on the use of a particular evaluation study, but more generally on how EU evolved over time, as the evaluation strategy was developed and more than 15 evaluation studies (Box 1) were conducted. For the present study, we employed an adapted version of Alkin and Taut’s [33] conceptualization of EU to ensure its diverse manifestations were identified. In their model, EU is either findings use (instrumental, conceptual, legitimative) or process use (instrumental, conceptual, symbolic). ‘Instrumental use’ involves direct use of evaluation-based knowledge for decision-making or for changing program practices [33]. ‘Conceptual use’ refers to indirect use of knowledge that leads to changes in the intended user’s understanding of program-related issues. ‘Symbolic use’ relates to situations in which those requesting the evaluation simply seek to demonstrate their willingness to undergo evaluation for the sake of reputation or status [29, 33]. Lastly, ‘legitimative use’ occurs when evaluation findings are used to justify previously undertaken actions or decisions [33]. We adapted Alkin and Taut’s [33] conceptualization by integrating its symbolic and legitimative uses under the broader concept of ‘persuasive use’ to also account for what Estabrooks [34] described as using evaluation as a persuasive or political means to legitimize a position or practice. Leviton and Hughes [35] further clarify the interpersonal influence that is integral to persuasive use, explaining that it involves using evaluation-based knowledge as a means to convince others to subscribe to the implications of an evaluation and hence to support a particular position by promoting or defending it. We added this term to stress the point made by previous authors that persuasive forms of EU can also serve constructive purposes [35, 36]. For instance, empirical evidence can be used persuasively to advocate for equity in global health. Symbolic and legitimative EU are terms that commonly carry negative connotations and are not easily applied to such constructive purposes. Persuasive use is included to draw attention to the different and concurrent ways in which evaluations can be used to influence reputations, judgment of actions or political positions.

Some examples may help clarify these different forms of EU. For instance, discussions during the evaluation process about the lack of potable water in a given village could lead intended users to think about strategies to bring water to the village; they might also recognize how helpful evaluations are in highlighting water needs for that village and how hard village locals have been working to fetch their water. These are forms of ‘conceptual process use’, in that intended users’ conceptions changed as a result of discussions during the evaluation process. Had such conceptual changes occurred as they learned of evaluation findings, this would have been ‘conceptual findings use’. Had intended users come to meet with locals and/or decided to dig a well, this would illustrate ‘instrumental process use’. It would have been ‘instrumental findings use’, had this decision to build a well been taken based on findings showing, for example, high morbidity rates associated with dehydration. Having already taken the decision to build the well, stakeholders could ask for an evaluation solely to empirically demonstrate the need for a well; this would be ‘legitimative use’. Or, they could have their well-building intervention evaluated without any intent or effort to use evaluations, but simply for ‘symbolic use’, to demonstrate their willingness to be evaluated. Then again, the well-building intervention could also undergo evaluation to provide convincing data that could be used in political claims advocating for human rights to potable water policies, thereby constituting ‘persuasive use’.

## Methods

### Research design

This evaluation used a qualitative single case study design and a descriptive approach to examine EPs’ discourses about EU over time [37, 38]. This was an instrumental case study, in that HELP’s evaluation strategy was chosen for its ability to provide insight into EU [39]. To document the evolution of EU over time, two waves of data collection were conducted by the first author in Burkina Faso using a diachronic approach with an interval of 29 months (July 2009 and November 2011). The 2009 data collection lasted 5 weeks and employed individual interviews. The 1-month 2011 data collection involved individual interviews as well as one group interview. Documentation and non-participatory observation provided contextual complementary information.

### Recruitment procedures

Objectives and procedures of the present study were explained to EPs upon soliciting their participation. When EPs responded positively, interviews were scheduled at a time and place of their convenience. Recruitment for individual interviews in 2009 and 2011 followed two purposeful sampling strategies [40]. The intensity sampling strategy (targeting persons intensely affected by the studied phenomenon) led us to recruit the principal evaluator and the NGO’s head of mission as the first participants [40]. Thereafter, the snowball sampling strategy was used, in which participants were asked to suggest other information-rich respondents. A conscious effort was made to limit the risks of ‘*enclicage*’ (a French term describing the risk that the researcher would be assimilated into a given clique and estranged from other groups and/or the larger group as a whole), as cautioned by Olivier de Sardan [41]. The extensive experience in the study context of one of the authors helped avoid such potential sampling biases. Data triangulation was also achieved by recruiting multiple participants with diverse relationships to HELP’s evaluation strategy as a means of obtaining varied perspectives and enhancing the study’s validity [42]. Such intra-group diversification was a conscious attempt to collect multiple viewpoints for a comprehensive appreciation of EPs’ individual and collective experiences [43, 44].

### Participants, data collection instrument and protocol

Semi-structured individual interviews were conducted in 2009 (n = 32; 15 respondents, 17 interviews) and in 2011 (n = 36; 17 respondents, 19 interviews) in Ouagadougou, Dori and Sebba. In each round of data collection, an extra interview was conducted with two EPs who had been particularly active and involved in the evaluation strategy and had more to say after a single interview; hence, the number of interviews exceeded the number of respondents by two in both collections. Table 1 presents the distribution of respondents for both data collections. Six EPs were interviewed in both 2009 and 2011. All EPs from HELP involved in the evaluation strategy were interviewed at least once, either in 2009 or 2011. EPs interviewed only in one data collection were either not working with HELP or out of the country during the other collection. Length of collaboration in the evaluation strategy ranged from three to 52 consecutive months for 16 EPs and was intermittent for the others. Eighteen EPs were locals from Burkina Faso, three were from West Africa, and five were international expats. Five were women, three held management positions, one was an evaluator, and another was a community outreach worker.

**Table 1:** Distribution of evaluation partners

Evaluation partner	2009		2011		Total^a^	
	n	Interviews	n	Interviews	n	Interviews
External evaluator	5	6	5	6	9	12
HELP Staff NGO requesting evaluation	6	7	6	7	8	14
Ministry of Health	3	3	3	3	5	6
ECHO representative Funding agency Advocacy partner	1	1	3	3	4	4
Total	15	17	17	19	26	36

^a^Evaluation partners in both 2009 and 2011: External evaluator: 1; HELP Staff: 4; Ministry of Health: 1; Total: 6

ECHO, European Commission’s Humanitarian Aid and Civil Protection department

Individual interviews lasted an average of 60 minutes. Interviews (individual and group) were semi-structured and followed an interview guide flexibly enough to allow it to evolve as the study progressed [40]. Questions were open-ended and solicited descriptions of EPs’ experiences and perceptions, as they had evolved over the course of the evaluation strategy, of (1) the evaluation strategy; (2) evaluation use; (3) collaboration with other EPs; and (4) the influence of evaluation upon them, other partners and their work environment. For most EPs, questions focused on the years 2009 to 2011, but those who had collaborated in the Niger evaluation were also free to recall their experiences starting in 2007. Specific examples of interview questions are presented in Box 2.

**Box 2** Interview guide: examples of questions

**Table:** 

2009 and 2011	What are your perceptions and experiences concerning: 1) The evaluation strategy - How did HELP’s evaluation strategy begin? - What activities were planned, realized? What were the effects observed? - When and how did you begin to collaborate in the evaluation strategy? - In which evaluation did you participate? How were you involved? - How do you feel about the way the evaluations went? Are there things you appreciated or things you did not like about the way the evaluations went? 2) Using evaluation - Among the evaluations in which you participated, which ones struck you as having something of interest? How so? - Were some of the evaluations useful? How so? Were some not useful? How so? Examples? - Were some of the evaluations used? How so? - Did you or other evaluation partners (EPs) gain something from participating in an evaluation activity? 3) Collaborating with other EPs - How would you describe the collaboration among evaluation partners? 4) Observed influences of evaluation upon yourself, other EPs and your work environment - Did you or your partners learn anything during the evaluations or from the evaluators? How so? - How have evaluations influenced you, your work? - What are the pros and cons of conducting evaluations at HELP? - What place does evaluation have at HELP? What place do you think it should have at HELP?
2011	- Since 2009, have you noticed changes in the evaluation strategy? How so? - How would you describe the state of the evaluation strategy now? - Have you noticed changes over time in the way evaluations were used? How so? - How would you describe the way evaluation partners have collaborated over time? - What challenges and successes have you noted about the evaluation strategy and the collaboration? - Over time, have you noticed different ways in which evaluation influenced you and/or the work and dynamics at HELP?

The group interview was conducted at the start of the 2011 data collection period before the individual interviews, as a means of discerning interpersonal dynamics and spurring collective brainstorming on the general questions of the present study; it lasted 90 minutes. This was a small group (n = 3; a manager and two coordinators) of HELP personnel who had been responsible for evaluation-related activities. Inspired by Kitzinger’s [45, 46] suggestions for focus groups, we used open-ended questions to foster interactions among them as a means of exploring emerging themes, norms and differences in perceptions regarding the evaluation strategy, EU and interpersonal dynamics among EPs. They were encouraged to explore different viewpoints and reasoning. Significant themes were later discussed in the individual interviews.

Interviews were conducted in French (Box 2), recorded digitally, transcribed and anonymized to preserve confidentiality. Transcripts were the primary data source for analyses.

Two additional sources of information provided insight into the study context, although not formal study data. Non-participant observation shed light upon EPs’ interpersonal dynamics and HELP’s functioning, as the first author spent 4 weeks during each of the two data collections in HELP’s offices interacting with HELP staff and with visiting partners. In 2011, she also accompanied HELP staff from all three sites on a 5-day team trip, during which a team meeting was held. Documents relevant to the evaluation strategy (e.g. evaluation plans and reports, scientific articles, policy briefs, meeting summaries, emails between EPs, advocacy documentation) were also collected to deepen understanding of the study’s context. These data provided opportunities for triangulating data sources, thereby strengthening the validity of EPs’ discourses.

### Analyses

Qualitative thematic content analyses were performed on the interview transcripts [47] using a mixed (inductive and deductive) approach and codebook. Coding and analysis were facilitated by the use of QDA Miner data analysis software. An adapted version of Alkin and Taut’s [33] model was used to identify and code different forms of EU. We used their conceptualizations of instrumental and conceptual EU but adapted the model, as mentioned earlier, by adding persuasive EU as a broad term encompassing the concepts of symbolic, legitimative and advocacy forms of EU. A specific code entitled ‘change’ was also created to capture any observations of changes related to EU mentioned and discussed by respondents in the 2011 interviews. For example, if a respondent in 2011 noticed that more evaluations had been conducted and disseminated and that this had led to more instances of EU, the code ‘change’ was applied to this sentence and integrated into the 2011 analyses and results (described below). Special attention was paid to ensuring that a broad range of EUs would be detected. After coding, we retrieved each type of EU and examined the coded excerpts for 2009 and for 2011 separately to identify and describe any apparent differences emerging from the respondents’ discourses on EUs between 2009 and 2011. In this manner, a thematic conceptual matrix was created, facilitating the organization and analysis of specific instrumental, conceptual and persuasive (including symbolic/legitimative) uses of evaluations in both 2009 and 2011. A summary of this matrix is presented in Table 2 [47]. The first author performed all the coding and analyses but met twice with a qualitative research consultant, six times with a co-author, and 10 times with a research colleague to discuss and verify the codebook and to ensure coding consistency and rigour over time (coding conferences). The iterative analysis process allowed for review of coded excerpts and hence continuity of the coding and interpretations. Attention was paid to capturing EPs’ interpersonal dynamics, as well as their individual and collective experiences over time [45, 46]. As mentioned, both non-participant observation and documentation helped the first author gain a deeper understanding of HELP’s context, but neither was analyzed systematically, due to lack of time and because interview data were already abundant. Analyses were not systematically validated by a second researcher, but two EPs active in the evaluation strategy commented on and validated a draft of the present article. The research was approved by the Ministry of Health of Burkina Faso. Ethical approval for the study was granted by the Research Ethics Committee of the University of Montreal’s Faculty of Arts and Sciences and by the Health Research Ethics Committee of the Ministry of Health of Burkina Faso.

**Table 2:** Use of evaluation findings and processes for 2009 and 2011

EPs	Findings use
	Instrumental findings use 2009
HS, EE	Avoid previous pitfalls
HS	Identify program malfunctions
HS, MoH, EE	Identify and implement locally sound and applicable solutions
HS, EE	Prepare and disseminate presentations, proposals, articles, policy briefs
	Instrumental findings use 2011
HS, AP	Improve practices and take decisions
HS, MoH	Identify, explain and plan for certain situations
	Conceptual findings use 2009
HS	Provide external perspective on the program and its effects
HS, MoH	Understand program malfunctions
	Conceptual findings use 2011
HS, AP	Understand effects of exemption and validate its mission
HS, MoH	Validate exemption program and boost team motivation
HS	Change perceptions of exemption program and of program evaluation
MoH, ER	Foster understanding of field reality and clinical data, cultivate a proactive and inquisitive approach
	Persuasive findings use 2009
HS, MoH	Validate exemption, promote collaboration with regional MoH
	Persuasive findings use 2011
HS, ER, AP	Support advocacy strategy to persuade policymakers and funders
HS, EE, ER	More persuasive use needs to target politicians
EP	Process use
	Instrumental process uste 2009
HS, EE, MoH	Improve evaluation appreciation and influence decisions (seeking funds, evaluation design, beneficiary criteria)
HS	Cultivate new collaborations and networks
	Instrumental process use 2011
HS, EE, MoH	Enhance EPs’ networks, promote partnerships and evaluation activities
MoH	Foster inclusion of usual outsiders to engage and resolve challenges
	Conceptual process use 2009
HS, MoH, EE	Facilitate capacity building (e.g. evaluation methodological, conceptual, technical understanding; humanitarian health field)
HS	Prompt curiosity and reflexive attitude; boost confidence
HS, EE, MoH	Facilitate communications increasingly tainted by scientific values and expectations
EE	Prompt cognitive changes facilitating evaluation use
	Conceptual process use 2011
HS, EE, MoH	Increase interest and capacities in exemption and in program evaluation
HS, EE	Change logic fostering receptivity, ability and yearning for scientific rigor

AP, Advocacy partner; EE, External evaluators; EP, Evaluation partner; ER, ECHO representative; HS, HELP Staff; MoH, Ministry of Health.

### Verification

Member checking was undertaken at various times and with different EPs to strengthen the validity of the findings [44]. For example, during data collections, the first author frequently verified her comprehension of the issues raised by EPs either during the interviews or after. The different themes emerging from analyses were discussed with several respondents to see whether they reflected EPs’ experiences and whether additional themes should be included. Drafts of the articles were sent by email to four participants who were thought to be most likely to have the time to read and comment on the drafts; two were able to respond to these member checking calls. Their feedback was always integrated into the iterative analysis process and usually also into the article drafts. Such member checking took place in informal discussions, during interviews and even in email correspondence. Other strategies were used to ensure responsiveness, sensitivity and reflexivity in the researcher’s approach and to support the validity of the present study [48]; these included co-coding and code discussions with a peer, using an iterative process in the analyses, peer debriefing (discussing the research methodology and analyses with academic peers), and keeping a log book of questions, ideas, challenges and decisions related to the study [49, 50].

## Results

We first present results on use of evaluation findings for 2009 and 2011, followed by results on use of evaluation processes for 2009 and 2011. In the 2011 interviews, respondents frequently mentioned EU examples similar to those presented in 2009. For the sake of brevity, we present only the examples from 2011 that cover new ground. Results are summarized in Table 2; it should be noted that the column on the left lists respondents speaking about use by intended users; hence, when external evaluators (EE) are indicated, it refers to themes discussed by evaluators about intended users’ EU, and not their own.

### Use of evaluation findings in 2009 and 2011

#### Instrumental use of evaluation findings

In 2009, participants described various ways in which evaluation findings were used instrumentally. An evaluator was pleasantly surprised by HELP’s interest and proactivity in implementing recommendations from a previous evaluation in Niger in 2007 (Box 1: study 9): “*They took our recommendations into consideration and completely changed their practice and the way they intervened*” (EE3). A HELP staff member corroborated this affirmation and described how they used evaluation findings to plan the exemption in Burkina Faso, paying specific attention to avoiding mistakes underscored in the previous evaluation report [51]. For example, as recommended by evaluators, HELP sought the collaboration of the DRS and MCDs – as representatives of the Ministry of Health (MoH) –right from the start of the user fee exemption program in Burkina Faso instead of setting up its intervention in parallel to the State’s health system, as had unwisely been done in Niger. EPs also noted that evaluation findings had helped them identify and resolve problems in their program and its implementation. For example, a HELP staff member recalled learning about preliminary evaluation findings (Box 1: study 7) that indicated some intended beneficiaries did not know they could be exempted from user fees. In response, HELP increased its awareness-raising efforts through radio information sessions and pamphlets. EPs also spoke about how evaluation findings had been used to identify solutions that were concrete, locally meaningful and applicable. According to a HELP staff member and MoH representatives, some findings were not used immediately but guided planning and decision-making. For example, following the presentation of an action research report (Box 1: study 15, Dori), MoH representatives decided to incorporate the recommendations into the district’s annual plan to set as priorities to improve health services quality and raise awareness of the exemption.

The 2011 interviews revealed that findings were being used for similar purposes as in 2009, including to improve practices and to guide decisions. For example, three HELP staff members referred to evaluation findings that had helped them better identify, select and recruit eligible beneficiaries (Box 1: studies 6 and 14). In that study, findings highlighted that, while indigents were a target group of the exemption, little had been done to reach out to them. This led HELP staff to test and use an effective selection strategy for indigents. Additionally, findings showing that the cost to exempt indigents was lower than expected led to a decision to increase the number of indigent beneficiaries for each health centre. Another use noted by an EP was that evaluation findings validated their decision to advocate for free healthcare, which enabled HELP to pursue its actions in this direction. Participants noted that evaluation findings were also used to identify, explain and resolve certain challenges they encountered. For instance, HELP staff recalled findings from study 7 (Box 1) showing that some intended beneficiaries were being deceived by health centre staff into paying user fees. This valuable information was used to resolve the problem by investing in efforts to raise awareness about the exemption program, its services, target beneficiaries and criteria. Another example concerned findings that demonstrated medical staff were complying with and respecting norms for medical prescriptions, contrary to rumours that they had been issuing excessive and inappropriate prescriptions since the exemption for personal gain. This valuable information guided the responses of the medical supervisors in the field, who were reassured to learn they did not need to worry much about this issue. Findings from another evaluation on workload (Box 1: study 16) suggested that, while the exemption program did increase the medical staff’s workload, it did not correspond to WHO’s definition of work overload [52]*.* An MoH representative noted that these findings had helped him to organize and manage his health centre’s resources, motivate his healthcare staff, and better adapt to the increase in consultations. An MoH representative also said evaluation findings were used to acknowledge accomplishments, review objectives, and correct practices when necessary. A HELP staff member correctly noted that changes in their practices (instrumental use) were preceded by changes in awareness (conceptualization).

### Conceptual use of evaluation findings

In 2009, respondents described a few instances of conceptual use of findings. One useful aspect of evaluation findings was that they provided the HELP staff with another, more external perspective. For example, one staff member observed that, at HELP, “*we have an internal vision because we work inside it*” and that evaluation findings (Box 1: study 12) could shed light on their partners’ views on various issues, such as when reimbursements for medical fees arrived late. HELP staff knew the reasons for this delay were outside their control, but “*it was interesting to see how the others* [partners] *perceived and sometimes criticized this; some even said it was because HELP was too late with reimbursements*” (HELP Staff (HS) 4). Similarly, a funding agency representative suggested that evaluation findings gave the agency a better understanding of people’s reactions to the exemption and, hence, of the field reality. Another EP suggested that findings pointed to deficiencies in the exemption program and were helpful in reflecting upon potential solutions: “*In my opinion, evaluations gave us a lot of experience and lessons to learn from*” (HS10).

In 2011, various EPs described how learning of the evaluation findings gave them a better understanding of the impacts of their work and of the exemption program. A HELP staff member recalled findings (Box 1: study 7) demonstrating that user fees were the primary barrier to healthcare accessibility, above and beyond geographical and cultural factors. Such findings validated the exemption program’s mission and counteracted previous arguments against user fee exemptions. Many of the findings also revealed positive effects of the exemption program on, for example, health service use. Consequently, another benefit of evaluation findings was that they boosted EPs’ motivations for their work:

“*I think this study* [Box 1: study 3] *was really useful and it had pretty important impacts on us. Speaking of the effects on the community, that was a motivating factor for us, it enabled us to see that by going in and out of the community all the time, we were actually bringing something*” (HS22).

After evaluation reports were presented, an MoH representative noted that he felt more capable when examining the health centre’s clinical data or even dealing with his patients after hearing about the different findings. One EP explained how some findings had changed his conception of the exemption and of program evaluation. He realized evaluations could detect the multiple effects of interventions, including some unexpected ones. For example, findings revealed that mothers felt empowered since the exemption implementation, as they could consult without their husbands’ approval and money [53]. Another participant also observed that hearing about evaluation findings changed many EPs’ receptivity to program evaluation. EPs were more forthcoming and followed evaluation activities better after attending report-presentation workshops (French: *ateliers de restitutions*) and hearing about the different evaluation findings. He recalled health workers saying, “…*the evaluators* ‘*come take our data and leav*e*!’ but after attending report-release workshops, they understood the findings and their utility; it encourages them to collaborate*” (HS2). Participants also believed evaluation findings enhanced their capacities and their understanding of the field reality.

### Persuasive use of evaluation findings

In 2009, persuasive use of evaluation was alluded to by EPs describing how evaluations supported their advocacy work. HELP staff said HELP’s major challenge was to disseminate evidence and convince their partners. Another explained their advocacy strategy, which involved partnering with the regional MoH (DRS and MCDs) and having them disseminate evaluation findings at national MoH meetings. One participant observed that Burkina Faso’s political decentralization facilitated the participation of the regional and district level MoH representatives, since they did not need consent from their national counterparts. The overarching goal was to convince policymakers of the benefits of user fee exemptions. HELP staff and MoH EPs suggested that the evaluation strategy validated their exemption work and bolstered their advocacy: “*We hope that maybe, with the expected results, a funding agency* […] *perhaps even the State, can participate* [in the exemption]”. Hence, HELP used findings persuasively to try to convince regional and national politicians to support and scale up the exemption in Burkina Faso. One EP noted that findings were used in project proposals and reports as a means to convince others of the worthiness of pursuing HELP’s exemption program.

In the 2011 interviews, EPs also spoke of using evaluation findings to influence partners and policymakers. HELP staff recalled partnering with University of Montreal researchers to produce and compile evidence on HELP’s exemption program. Their studies demonstrated the value of the exemption, thereby establishing the pillars of HELP’s advocacy work. Evidence suggested that lifting the financial barriers to health access was commendable and logical. HELP staff recalled presenting findings to the MoH at national and international conferences to promote adoption of a national exemption program. Some also spoke about partnering with Amnesty International to advocate for evidence-based policymaking by the State [24]. HELP frequently shared scientific documentation with its funding agency, advocating for a national exemption program. An evaluator acknowledged HELP’s limited success in convincing politicians to adopt and scale up the exemption program, which sometimes led HELP and its partners to question “*…the use of all our work?*” (EE8). He explained how HELP and the evaluation strategy’s decision-makers had opted to end the evaluation strategy activities gradually, as it had already produced sufficient knowledge on essential questions, and to focus instead on HELP’s advocacy to find ways to increase politicians’ use of scientific evidence. Funding agency representatives criticized HELP’s persuasive use, suggesting that HELP needed to be more proactive in its advocacy strategy to seek and seize every diffusion opportunity:

“*I have the impression that HELP doesn’t really know how to show the value of its research* […] *Diffusion activities were good but I think they could have done even better. One example is the last diffusion activity; they weren’t able to meet with the Ministry of Health, even though this is a key stakeholder*” (ECHO representative)*.*

Meanwhile, HELP staff suggested that further targeting diffusion efforts to community members would benefit the exemption program’s activities. One difficulty with this, alluded to by an MoH representative, was the necessity of translating many of the presentations into local languages, as many in the community did not speak French. An evaluator explained how financial constraints led to the prioritization of knowledge transfer (KT) activities targeting political leaders, in hopes this would produce greater impacts. Nevertheless, he explained how evaluators with HELP had sought creative means, such as policy briefs and short films, to reach a diverse audience, focusing particularly on policymakers.

In both 2009 and 2011, one challenge underscored by EPs was that of interesting policymakers in these evidence-based findings and in the exemption itself. In 2009, the discourse was hopeful, while the 2011 interviews expressed more disappointment and doubt regarding the feasibility of advocacy objectives. From the 2011 interviews, it was clear that HELP had used evaluation findings to try to persuade others of the value of the exemption program. Whether they succeeded in their persuasive attempts is another interesting question, distinct from the present article’s focus specifically on EPs’ own use.

Overall, EPs described instances of instrumental, conceptual and persuasive use of findings in both 2009 and 2011. However, they discussed using more evaluations in 2011 than in 2009. One evaluator asserted that there was so much more EU by EPs in 2011 that it was not comparable to 2009. An evaluator also suggested this was because only one study, along with the action research project, had been finalized by the time of our first data collection in 2009. EUs were also described in greater detail by EPs in 2011 than in 2009.

### Use of evaluation processes in 2009 and 2011

#### Instrumental use of evaluation processes

Recommendations are often associated with findings, as they are frequently presented in the final evaluation report. However, in 2009, EPs recalled various lessons already learned during the evaluation process. For example, HELP staff recalled having discussions with evaluators and pointing out a problem, which was that the eligibility criterion for HELP’s user fees exemption for breastfeeding mothers was too vague, because breastfeeding duration varies widely across mother/baby pairs (Box 1: study 13). Based on discussions during the evaluation process, HELP stakeholders operationalized mothers’ eligibility to 2 years following a baby’s birth, and this information was then shared via guidelines disseminated to all health centres. Further, EPs who had been involved in the 2007 evaluation in Niger (Box 1: study 9) recalled learning that, because the evaluation had only been organized near the end of the project, it was not possible to use a pre–post design, which would have been the most meaningful methodologically. Having learned from this experience, HELP coordinators consulted the evaluator while planning their Burkina Faso exemption program to ensure pre–post designs could be used in the evaluations to measure the program’s efficacy more reliably. The coordinators had worked both in Niger and then in Burkina Faso and, hence, carried over such lessons. An evaluator recalled how his being consulted at the beginning of the Burkina Faso program led HELP stakeholders to delay implementing the exemption there in order to collect baseline data, despite the ethical dilemma that delaying the exemption meant delaying saving lives. Process discussions clarified that, irrespective of when the exemption would be implemented, the duration of the program was fixed and therefore the number of lives saved in the given time frame would be identical. Moreover, if careful planning led more convincing evidence of the exemption’s beneficial effects, HELP’s advocacy would have greater persuasive power. It was also made clear that funding a series of evaluations could produce useful knowledge for advocacy. Stakeholders made use of these discussions and decided (instrumental process use) to seek funds from a funding agency. They received funding to develop the evaluation strategy, which evolved over time into an extensive series of evaluations. New collaborations and networks with different African institutions were also born out of this initial evaluation partnership.

In 2011, an evaluator suggested that the initial collaboration process between HELP and evaluators had stimulated a proliferation of partnerships and networks among EPs, which developed further into their own respective documentation and advocacy projects. An MoH representative reported having learned a great deal about writing research protocols while collaborating with the external evaluators, which subsequently led him to write his own internal research protocol. Another MoH representative also recalled an evaluation of obstetric service use in which community members were, to his surprise, stakeholders in the research process even though they had little education (Box 1: study 8). He quickly realized the added value of their participation, as they gradually understood and supported the findings, became more proactive than usual, and identified sensible means of increasing obstetrical service use. Another instrumental use described by an evaluator and an MoH representative was that their collaboration may have sparked some EPs’ interest and motivation to develop their capacities further, as several subsequently chose to pursue graduate studies in health research. The evaluator believed that, for some EPs, the experience of networking with researchers and developing new contacts with local and international supervisors may have facilitated admissions to graduate schools and scholarships.

### Conceptual use of evaluation processes

In the 2009 interviews, HELP staff described experiencing capacity building during evaluations and said their methodological, conceptual and technical understanding of the different research phases had been reinforced or updated. A HELP coordinator suggested his comprehension of public health had also improved during evaluations, which aided his management of the NGO. Other conceptual changes were noted. As another HELP staff member explained, “*What was good was that we were participating and engaging* [in the evaluations] *so it was not something external that just fell upon us… the fact that we had to ask questions meant we had to think about it*” (HS2). Through this process, they realized they could ask pertinent questions that strengthened their confidence. One HELP staff member said that participating in evaluations sparked a “*spirit of curiosity*” necessary to ask research questions and stimulated a sense of agency in pursuing answers. He believed more needed to be done to maintain such capacities and make the staff more autonomous. Another HELP staff member described how EPs’ interactions facilitated discussions and fostered the development of a common vocabulary infused with values such as scientific rigour and evaluation use. An evaluator believed evaluation processes had also led to the harmonization of EPs’ perceptions of the exemption and its impacts.

In 2011, EPs conveyed numerous examples of conceptual process use, including capacity building in evaluation (conceptualization, application and practice). An evaluator reported improvements over time in many of the HELP staff’s research, professional and management skills. One HELP staff member said working closely with evaluators was a source of inspiration, guidance and feedback that made him feel stronger and supported. Some reported that participating in evaluations helped their thinking become more rigorous, gave them another perspective on the program, highlighted the importance of measuring program effects and heightened their receptivity to evaluation. Another HELP staff member noted that it was when EPs really got involved in evaluations that they began to understand the findings and the value of evaluation, which in turn facilitated integration of EU into the HELP organization. HELP staff member said that participating in the evaluation dissemination process had many benefits, because the preparation and interactions involved required them to reflect more actively on the findings, which, in turn, enhanced their assimilation of the findings, making those more applicable. In his opinion, evaluation processes deepened and harmonized partners’ understanding of the exemption program, helping them find a common direction. A HELP coordinator also said, “*By rubbing shoulders with the evaluation culture, we were won over!*” (HS7). He described staff as being more prudent in their communications, using language that was measured, succinct, goal-oriented, scientific and evidence-based: “*It prevents us from arguing over facts that are not backed up*” (HS7). Another HELP staff member learned that precise communication with evaluators was helpful in obtaining results in tune with his information needs. An EP explained how the evaluation strategy expanded their professional networks, which facilitated information sharing and knowledge transfer. For all these reasons, various respondents believed other humanitarian NGOs involved in emergency action would also benefit from documenting the effects of their work.

Descriptions of conceptual process use examples changed between 2009 and 2011 as EPs suggested they had learned a great deal about evaluation, which changed their attitudes and behaviour with regard to evaluation activities. In 2011, respondents had more to say and were more enthusiastic about sharing the changes in their work, attitudes and understanding brought on by evaluation. Conceptual use appeared to have increased over time. Looking back over the evolution of the strategy, an evaluator highlighted the fact that the first evaluation activities, which proved useful for HELP, opened the way for more and progressive development of the evaluation strategy as new funding was granted for each successive phase of the exemption project. In 2009, EPs were impatient to hear about the evaluation findings, but once the evaluations were completed and the results shared, EPs became much more receptive to evaluators and convinced that program evaluation was pertinent for HELP. The evaluator pointed out that, as evaluation questions were answered, more were raised, and the evaluation strategy team developed progressively more evaluation activities. This was corroborated by documentation produced and shared by the evaluation strategy team. Thereafter, EPs used evaluation findings more frequently and EU became progressively mainstreamed into HELP’s exemption program.

### Persuasive use of evaluation processes

In both 2009 and 2011, no respondent described any form of persuasive process use. In no instance did EPs describe having engaged in the evaluation process simply to satisfy the wish of their funding agency, to promote their own reputation or to convince others. As noted earlier, some spoke about engaging in the evaluation process, but their focus was more on using the findings than on the evaluation process itself.

The 2011 interviews shed light on the dynamics between some HELP staff and evaluators that inevitably influenced evaluation processes and perhaps EU. While these conditions influencing EU are a topic of their own to be covered in a future article, a few details provide valuable insight into the present study findings. For example, participants suggested that some HELP staff were reluctant to participate in the evaluation process partly because they did not completely trust the motives of evaluators who, according to them, may have been more concerned about furthering their research careers than about HELP’s actual mission. They expressed their discomfort to colleagues and to evaluators, but did not object to the conduct of evaluations and, in the end, found them useful.

As described in the methods section, non-participant observation and documentation provided valuable contextual information on the evaluation strategy and EPs. While systematic analysis of these data was not feasible due to time constraints, both sources provided relevant information. Non-participant observation enabled the first author to become immersed in the study context, to detect welcoming, collaborative and friendly dynamics between most EPs, and to observe that EPs were generally at ease in communicating with each other about questions and concerns. Certain other dynamics were also apparent, such as the relatively peaceful and friendly interactions between HELP staff and EPs. HELP staff tended to joke, tease one another, and laugh together. They had social gatherings on evenings and weekends. It was also apparent that some HELP staff tended to have more affinity than others with evaluators. All evaluators were warmly welcomed by HELP staff. While reluctance to trust evaluators’ motives was discussed only in individual interviews, informal discussions revealed that these issues had been discussed explicitly in team meetings. Team meetings appeared to foster frank and direct communication. Even so, various participants mentioned that, in Burkina Faso, anyone dealing with politics learns to communicate using a “*langue de bois*”, a diplomatic way of avoiding discussing sensitive issues directly, and this was indeed observed in interviews and interpersonal dynamics.

Collected documentation relating to the evaluation strategy and to collaborations among EPs also helped the first author become immersed in the working dynamics of EPs. It corroborated EPs’ discourses about increasing efforts over time to formalize agreements together by documenting contracts, report presentations and collaboration plans. Documents relating to evaluation activities and results (e.g. reports, scientific articles, policy briefs) proliferated between 2009 and 2011, supporting EPs’ descriptions of an increase in evaluation activities and EU over time. Emails between the principal evaluators and HELP coordinators were frequent from 2009 and too numerous to examine systematically, but generally their content demonstrated frank and transparent problem-solving, brainstorming and sharing of information about activities, events and scientific articles. As noted earlier, these forms of data were collected by the first author to complement the individual and group interview data and as a means of becoming better acquainted with the EPs’ working environment.

## Discussion

The present study enabled us to identify and provide rich descriptions of the different forms of EU in which EPs engaged between 2009 and 2011, as HELP’s evaluation strategy was rolled out. Descriptions of EU, including instrumental, conceptual and persuasive use of findings and/or processes, were generally more elaborate and specific in 2011, and EPs emphasized that EU had increased since 2009. EPs described all the forms of EU found in Alkin and Taut’s [33] categories, with the exception of persuasive (and symbolic) process use. Indeed, evaluation findings were used instrumentally by EPs for numerous purposes, including to identify program malfunctions and come up with solutions, to guide decisions and actions, and to manage and motivate colleagues. EPs also used findings conceptually in many ways, such as learning to see their program and work from an external perspective, recognizing the value of the exemption program and of their own work, communicating and motivating staff, and gaining an appreciation for the field reality and for program evaluation. EPs also used findings in a persuasive manner to convince others to support and scale up the exemption program. Persuading political decision-makers proved challenging, which corroborates Dagenais et al.’s [8] findings in the same national context and points to the common difficulty of making policymaking more evidence-based [54, 55]. It became clear by 2011 that scientific knowledge was abundant and accessible to anyone interested, and therefore the evaluators felt they had done their work. It had also become clear that, to conserve the scientific rigour and neutrality expected of university researchers, the principal evaluators had to rethink their involvement in advocacy activities. Negotiating where KT ended and advocacy began presented an interesting challenge for external evaluators, HELP coordinators and other EPs. Financial limitations also led to difficult decisions regarding what KT activities could be undertaken, by whom, and for whom.

Participating in evaluations also prompted many instances of process use. Overall, the evaluation process provided countless opportunities for EPs to reflect upon their program and how they worked together and interacted. It provided opportunities to develop partnerships, communicate problems, and identify and implement potential solutions. It was clear, however, that issues of mistrust regarding evaluators’ motives and the allocation of evaluation resources were still taboo for several participants and not discussed openly among EPs. This may have negatively influenced their collaboration. Finding ways to overcome such challenges might result in more successful collaboration, evaluation participation and EU. Nevertheless, evaluation activities led EPs to learn about their program, evaluation processes and research methodology. By engaging in evaluations and interacting with evaluators, EPs learned to think in a different way about programs and scientific rigour. Since Patton’s original work [56] on utilization-focused evaluations, which described the benefits of participatory approaches and process use, many authors have documented the importance of engaging participants in the evaluation process [5, 57–62]. The literature suggests that participation should ideally begin at conceptualization of an evaluation study [31]. While this may be ideal, the limited time and financial resources common to humanitarian practitioners, including in HELP’s organizational context, led some EPs to disinvest or invest only partially in the evaluation strategy. This was a source of frustration for evaluators and those more invested in the evaluation strategy. Yet, some EPs described how participating principally in the dissemination phase was helpful to them as a creative way of dealing with this issue of limited time, as it led them to invest in and reflect upon all the previous phases of evaluation that had led to the results they were mandated to present. This is an interesting option to consider when participating in all stages of all the evaluations is impossible, as it was for some EPs.

The reason for the absence of persuasive (symbolic) process use was not explained by our respondents, but Højlund’s [63] thoughts on an organization’s internal propensity and its external pressures to engage in evaluations provide interesting insights. More specifically, from the individual and group interview data, it was clear that, while HELP’s funders had requested the first evaluation, EPs felt little external pressure to undertake evaluations. The propensity to evaluate came from the inside, primarily from HELP’s coordinator, and the overall motives for evaluation were clear: to have credible findings to inform advocacy for accessible health services, and to learn about and improve the exemption program. Engaging in an evaluation process for symbolic reasons simply did not seem to be a concern for EPs. Respondents intended to use the evaluation findings, but not the process, for persuasive purposes.

A frequent challenge during the present study was to determine what exactly sparked EU. For instance, in the section above on instrumental process use in 2009, we discussed how evaluation discussions led participants to reconsider their approach and to seek more evaluation resources, develop the evaluation strategy, and form new collaborative networks and partnerships. It is difficult to pinpoint exactly when and why such attitude changes and decisions occurred. Were they prompted directly by discussions during an evaluation activity, which would clearly fall under process use, or did they arise simply from EPs being immersed in an evaluation strategy and thus in frequent interaction and communication with evaluators? This points to a limitation of the present study associated with respondents’ difficulty in recalling specifically what triggered a given decision or action. This issue was discussed by Leviton and Hughes [35], who described how, under such conditions, it is difficult to decipher where conceptual use ends and instrumental use begins and, in turn, to categorize use according to a specific EU taxonomy such as that of Alkin and Taut [33].

In the real-world setting of the present study, instrumental, conceptual and persuasive uses often overlapped and were not easily teased apart. Therefore, current EU taxonomy has received its share of criticism for operationalization challenges or for constraining the scope of evaluation consequences [64–66]. We encountered this challenge of limited scope when, for example, EPs discussed long-lasting effects the evaluation process had on them (e.g. expanded professional network, increased funding for the evaluation strategy). While we were sufficiently able to decipher the source of such effects so that we could categorize them using Alkin and Taut’s [33] EU taxonomy, it is true that Kirkhart’s [66] integrated theory of evaluation influence is better adapted to such situations. Kirkhart implored researchers to expand the scope of EU by acknowledging the full range of evaluation influences and suggested that existing conceptualizations of EU tend to overlook the value of process use and of uses that occur unintentionally or incrementally over time [66]. However, that model would also have presented its share of challenges, as our respondents were frequently unable to provide specific information about the source, intentionality or timeframe of influence, the three principal dimensions of the model. Providing such information was difficult for them, possibly because of the sheer number of evaluation activities undertaken as part of the evaluation strategy. We therefore concur with other authors in believing that Alkin and Taut’s [33] taxonomy of EU remains relevant [10], as we found that it facilitated our in-depth examination of the multiple facets and specific forms (instrumental, conceptual, persuasive) of EU processes and findings over time. We agree with Mark [67] that, rather than reinventing the wheel, a reasonable solution would be to see the concept of evaluation use not as competing with that of evaluation influence but rather as being complementary to it. This may help researchers, evaluators and intended users attend to an evaluation’s broad array of potential consequences when planning for, conducting or studying evaluations [67].

Another potential limitation of the study stems from the high mobility and turnover among participants, such that we were able to capture the evolving perspectives of only six EPs over the two data collections. Clarke and Ramalingam [68] discussed the fact that high turnover is common in humanitarian NGOs and presents both challenges (e.g. loss of organizational memory) and opportunities (e.g. bringing on new staff in line with evolving program objectives). Interviewing the same participants in both phases of the study might have produced different results, but the present findings reflect change processes that are common to the humanitarian sector reality. Patton [69] described turnover as the Achilles’ heel of utilization-focused evaluation and discussed the importance of working with multiple intended users so that the departure of one is not necessarily detrimental to EU. Such a challenge and solution apply to the present study, in which our aim was to follow multiple intended users who were present for either part or all of the study period. In fact, those interviewed in both data collections were four of the primary intended users (from HELP), an external evaluator, and an MoH representative. Hence, the study enabled us to examine the evolution of EU and how it was influenced by interpersonal dynamics and changing realities, such as turnover, that are common to many humanitarian NGOs, through the perspectives of EPs who had experienced the evaluation strategy in a variety of ways.

A third potential limitation of the study is that all three authors have, over time and to different degrees, developed professional and friendly relationships with various EPs – the second and third authors having acted as consultants for HELP; in a collaboration that evolves over time, this is not surprising and perhaps sometimes even desirable, but may make it difficult to maintain the neutrality required of an external evaluator. Mitigating these human dimensions while navigating the numerous potential evaluator roles, as described by Pattona and LaBossière [70], may have led to forms of normative discourse. Nevertheless, it is worth noting that the first author completed the research in total independence and without interference from HELP in the data. She undertook the study without payment and received only periodic material or logistical support from HELP when necessary to conduct the data collection. Also, only the first author, who never worked as consultant for HELP, conducted the interviews and analyzed and interpreted the data. While most evaluation studies have examined a single evaluation study or a specific evaluation program at one point in time [for examples see 10], the present study examined EU over time, with data collections separated by 29 months, and during an ongoing series of evaluation studies that were part of the evaluation strategy which originated from a single evaluation study in Niger in 2007. This was challenging because the literature provided few examples to guide the conceptualization and conduct of the present study. Yet, this was also the strength of the study, as it presented an innovative standpoint from which to examine EU. Future research may provide further guidance for the study of EU following a single evaluation or multiple evaluations embedded within an organization’s routine operations. Clearly, in our study context, evaluation partners’ EU evolved over time, and the study’s design enabled us to decipher the multiple forms in which EU occurred, including not only instrumental and conceptual forms of process and findings use, but also persuasive findings use. The study’s methodology was bolstered by our ability to seek out multiple groups of participants and thereby to triangulate perspectives. An important new contribution of the present study is, in fact, that it presents the views of both evaluators and intended users.

## Conclusion

In 2004, a report by WHO emphasized the need to enhance the use of empirical knowledge in the health sector [23]. The following year, WHO members pledged to achieve universal healthcare and again highlighted the importance of using empirical evidence to guide global health policymaking and practices [26]. Nevertheless, how exactly are evaluations performed and used in global health and humanitarian contexts? Henry [65] pointed out that most of the EU literature is theoretical or conceptual and that very little of it examines EU systematically. Sandison [9] and Oliver [71] described how empirical research on EU within humanitarian organizations is particularly rare. HELP’s user fee exemption program presented an opportunity to include an evaluation strategy to study and document the processes, challenges, successes and impacts of the program. Simultaneously, this evaluation strategy itself presented an exceptional occasion to study and understand how evaluations can be both useful and actually used in the humanitarian context. In examining EU resulting from HELP’s evaluation strategy, the present case study helps bridge the knowledge-to-action gap by shedding light on the different ways HELP and its partners used evaluations. By studying how they collaborated to infuse EU into their practice and by examining how their discourses on EU evolved between 2009 and 2011, we determined that they increasingly used evaluation processes and findings instrumentally and conceptually, and used evaluation findings persuasively. Such uses served the mission of HELP’s exemption program in numerous ways by, among other things, supporting its members’ ability to think critically, improving their collaboration, identifying problems in the program and potential solutions, facilitating decision-making, and supporting HELP’s advocacy activities. In March 2016, we learned that Burkina Faso’s Ministerial Council [72] announced that, by April 2016, a national policy would be implemented to provide free healthcare for children under five and pregnant women, and to give women free access to caesarean sections and deliveries as well as to breast and cervical cancer screenings. While numerous barriers remain between empirical knowledge and its uptake in the political arena, and while it seems particularly difficult to use pilot studies to inform public policymaking [21], there is little doubt that HELP’s pilot exemption program and its associated evaluation strategy and advocacy activities, along with the work of partner organizations, played an important role in inspiring Burkina Faso’s recent policies. In a subsequent paper, we will discuss our analyses of the conditions that appear to have influenced EU among HELP’s evaluation partners.

## Abbreviations

DRS: Directeur régional de la santé (regional health director)
ECHO: European Commission’s Humanitarian Aid and Civil Protection department
EP: Evaluation partner
EU: Evaluation use
HELP: Non-governmental organization *Hilfe zur Selbsthilfe e.V.*
KT: Knowledge transfer
MCD: Médecin chef de district (district chief physician)
MoH: Ministry of Health
NGO: Non-governmental organization

## References

[1] Darcy J, Knox Clarke P (2013). Evidence & knowledge in humanitarian action. Background paper, 28th ALNAP meeting, Washington, DC, 5–7 March 2013.

[2] Beck T (2003). Evaluating humanitarian action: an ALNAP guidance booklet.

[3] Crisp J (2004). Thinking outside the box: evaluation and humanitarian action. Forced Migration Review..

[4] Hallam A (2011). Harnessing the power of evaluation in humanitarian action: An initiative to improve understanding and use of evaluation. ALNAP working paper.

[5] Hallam A, Bonino F (2013). Using evaluation for a change: insights from humanitarian practitioners.

[6] ALNAP (2006). Evaluating humanitarian action using the OECD-DAC criteria: an ALNAP guide for humanitarian agencies.

[7] Harveu P, Stoddard A, Harmer A, Taylor G, DiDomenico V, Brander L (2010). The state of the humanitarian system: Assessing performance and progress. A pilot study. ALNAP working paper.

[8] Dagenais C, Queuille L, Ridde V (2013). Evaluation of a knowledge transfer strategy from a user fee exemption program for vulnerable populations in Burkina Faso. Global Health Promotion.

[9] Sandison P (2006). The utilisation of evaluations. ALNAP Review of Humanitarian Action in 2005: Evaluation utilisation.

[10] Cousins JB, Shulha LM, Shaw I, Greene JC, Mark M (2006). A comparative analysis of evaluation utilization and its cognate fields of enquiry. Handbook of evaluation: policies, programs and practices.

[11] Ridde V, Heinmüller R, Queuille L, Rauland K (2011). Améliorer l’accessibilité financière des soins de santé au Burkina Faso. Glob Health Promot.

[12] Ridde V, Queuille L, Atchessi N, Samb O, Heinmüller R, Haddad S (2012). The evaluation of an experiment in healthcare user fees exemption for vulnerable groups in Burkina Faso. Field ACTions Science Reports..

[13] Ridde V, Queuille L (2010). User fees exemption: One step on the path toward universal access to healthcare.

[14] HELP (2008). Annual Report 2008.

[15] INSD (2010). La région du Sahel en chiffres.

[16] World Health Organization (2007). World health statistics 2007.

[17] World Health Organization (2014). World Health Statistics 2014.

[18] Traoré C, Ridde V, Queuille L, Kafando Y (2012). Préface. Capitalisation de politiques publiques d'exemption du paiement des soins en Afrique de l'Ouest.

[19] Ridde V, Robert E, Meessen B (2012). A literature review of the disruptive effects of user fee exemption policies on health systems. BMC Public Health..

[20] Olivier de Sardan JP, Ridde V (2015). Public policies and health systems in Sahelian Africa: theoretical context and empirical specificity. BMC Health Serv Res.

[21] Ridde V (2015). From institutionalization of user fees to their abolition in West Africa: a story of pilot projects and public policies. BMC Health Serv Res.

[22] Ridde V, Queuille L, Ridde V, Queuille L, Kafando Y (2012). Capitaliser pour apprendre et changer les politiques publiques d'exemption du paiement des soins en Afrique de l'Ouest: une (r)évolution en cours?. Capitalisation de politiques publiques d'exemption du paiement des soins en Afrique de l'Ouest.

[23] World Health Organization (2004). World Report on Knowledge for Better Health: Strengthening Health Systems.

[24] International A (2010). Burkina Faso: Giving life, risking death. Time for action to reduce maternal mortality in Burkina Faso. Index number: AFR 60/001/2010.

[25] World Conference on Science (1999). Excerpts from the declaration on science and the use of scientific knowledge. Sci Commun..

[26] World Health Organization (2013). The World Health Report: Research for Universal Health Coverage.

[27] Ridde V, Diarra A, Moha M (2011). User fees abolition policy in Niger. Comparing the under five years exemption implementation in two districts. Health Policy.

[28] D’Ostie-Racine L, Dagenais C, Ridde V (2013). An evaluability assessment of a West Africa based non-governmental organization's (NGO) progressive evaluation strategy. Eval Program Plann.

[29] Shulha LM, Cousins JB (1997). Evaluation use: theory, research, and practice since 1986. Eval Pract.

[30] Herbert JL (2014). Researching evaluation influence: a review of the literature. Eval Rev.

[31] Patton MQ (2008). Utilization-focused evaluation.

[32] Patton MQ (2007). Process use as a usefulism. N Dir Eval..

[33] Alkin MC, Taut SM (2003). Unbundling evaluation use. Stud Educ Eval..

[34] Estabrooks C (1999). The conceptual structure of research utilization. Res Nurs Health..

[35] Leviton LC, Hughes EFX (1981). Research on the utilization of evaluations. Eval Rev.

[36] Weiss C, Weiss C, Lexington MA (1977). Introduction. Using Social Research in Pubic Policy Making.

[37] Yin RK (1999). Enhancing the quality of case studies in health services research. Health Serv Res.

[38] Yin RK (2014). Case study research: design and methods.

[39] Stake RE, Denzin NK, Lincoln YS (2003). Case studies. Strategies of qualitative inquiry.

[40] Patton MQ (1990). Qualitative evaluation and research methods.

[41] Olivier de Sardan JP (2003). L’enquête socio-anthropologique de terrain : synthèse méthodologique et recommandations à usage des étudiants Niamey.

[42] Creswell JW, Plano CV (2006). Designing and conducting mixed methods research.

[43] Pires AP, Poupart J, Deslauriers J-P, Groulx L-H, Laperrière A, Mayer R, Pires AP (1997). Échantillonage et recherche qualitative: essai théorique et méthodologique. La recherche qualitative: Enjeux épisémologiques et méthodologiques.

[44] Stake RE (2010). Qualitative research: Studying how things work.

[45] Kitzinger J (1994). The methodology of Focus Groups: the importance of interaction between research participants. Sociol Health Illness.

[46] Kitzinger J (1995). Qualitative research: introducing focus groups. BMJ.

[47] Miles MB, Huberman M (1994). Qualitative data analysis: an expanded sourcebook.

[48] Morse JM, Barrett M, Mayan M, Olson K, Spiers J (2002). Verification strategies for establishing reliability and validity in qualitative research. Int J Qualitative Methods.

[49] Patton MQ (2005). Qualitative research. Wiley Online Library.

[50] Ritchie J, Lewis J, Nicholls CM, Ormston R (2013). Qualitative research practice: a guide for social science students and researchers.

[51] Ridde V, Diarra A (2009). A process evaluation of user fees abolition for pregnant women and children under five years in two districts in Niger (West Africa). BMC Health Serv Res..

[52] Antarou L, Ridde V, Kouanda S, Queuille L (2013). La charge de travail des agents de santé dans un contexte de gratuité des soins au Burkina Faso et au Niger [Health staff workload in a context of user fees exemption policy for health care in Burkina Faso and Niger]. Bull Soc Pathol Exot.

[53] Samb O, Belaid L, Ridde V (2013). Burkina Faso: la gratuité des soins aux dépens de la relation entre les femmes et les soignants?. Humanitaire: Enjeux, pratiques, débats..

[54] Knox Clarke P, Darcy J (2014). Insufficient evidence? The quality and use of evaluation in humanitarian action.

[55] Crewe E, Young J (2002). Bridging research and policy: Context, evidence and links. Working Paper 173.

[56] Patton MQ (1978). Utilization-focused evaluation.

[57] Buchanan-Smith M, Cosgrave J (2013). Evaluation of humanitarian action: Pilot guide.

[58] Cousins JB, Leithwood K, Louis KS (1998). Organizational consequences of participatory evaluation: School district case study. Organizational learning in schools.

[59] Cousins JB, Kellaghan T, Stufflebeam DL, Wingate LA (2003). Utilization effects of participatory evaluation. International handbook of educational evaluation: Part two: Practice.

[60] Cousins JB, Earl LM (1992). The case for participatory evaluation. Educ Eval Policy Analysis.

[61] King JA (2007). Developing evaluation capacity through process use. N Dir Eval.

[62] Patton MQ, Segone M (2008). Future trends in evaluation. From policies to results: Developing capacities for country monitoring and evaluation systems.

[63] Højlund S (2014). Evaluation use in the organizational context – changing focus to improve theory. Evaluation.

[64] Henry G (2003). Influential evaluations. Am J Eval.

[65] Henry G (2003). Beyond use: understanding evaluation's influence on attitudes and actions. Am J Eval.

[66] Kirkhart KE (2000). Reconceptualizing evaluation use: an integrated theory of influence. N Dir Eval..

[67] Mark MM (2011). Toward better research on—and thinking about—evaluation influence, especially in multisite evaluations. N Dir Eval.

[68] Clarke P, Ramalingam B (2008). Organisational change in the humanitarian sector.

[69] Patton MQ (1997). Utilization-focused evaluation.

[70] Patton MQ, LaBossière F, Ridde V, Dagenais C (2009). évaluation axée sur l'utilisation. Approches et pratiques en évaluation de programme.

[71] Oliver ML. Evaluation of emergency response: humanitarian aid agencies and evaluation influence. Dissertation, Georgia State University, 2008. http://scholarworks.gsu.edu/pmap_diss/23. Accessed 11 Jan 2016.

[72] Le Ministère du Burkina Faso. Compte-rendu du Conseil des ministres du mercredi 2 mars 2016. Portail officiel du gouvernement du Burkina Faso. Ouagadougou: Le Ministre de la Communication et des Relations avec le Parlement; 2016.

